# Knowledge of Antibiotic Resistance Among Dental Students at Teerthanker Mahaveer University: A Cross-Sectional Study

**DOI:** 10.7759/cureus.116876

**Published:** 2026-09-24

**Authors:** Nevidita Pertin, Ankita Jain, Pradeep Tangade, Vikas Singh

**Affiliations:** 1 Department of Public Health Dentistry, Teerthanker Mahaveer Dental College and Research Centre, Teerthanker Mahaveer University, Moradabad, IND

**Keywords:** antibiotic prescribing, antibiotic resistance, antimicrobial stewardship, dental education, dental students, knowledge and attitudes

## Abstract

Background

Antibiotic resistance is a serious global health problem driven in part by the misuse of antibiotics by healthcare professionals, including dentists. Identifying opportunities to improve dental students' knowledge is important for promoting rational and evidence-based antibiotic prescribing.

Objective

The objective of this study is to assess knowledge and misconceptions regarding antibiotic use and antibiotic resistance among dental students at Teerthanker Mahaveer University.

Methodology

A cross-sectional survey was conducted among fourth-year dental students and clinical interns (n=108). A validated questionnaire was administered using Google Forms. Data were analysed using SPSS version 24.0, and the chi-square test was used to assess the associations between demographic characteristics and knowledge level.

Results

The majority of participants were aware of the importance of completing the antibiotic course (n=99; 91.7%) and the role of antibiotic misuse in contributing to antibiotic resistance (n=90; 83.3%). However, (n=71; 65.7%) incorrectly thought that antibiotics could cure viral infections. Only 40 (37.0%) students were aware that antibiotic-resistant bacteria can be spread from person to person. Gender, academic role, and medical family history were not statistically associated with knowledge level.

Conclusion

Participants demonstrated a good understanding of antibiotic adherence and the role of antibiotic misuse in the development of antibiotic resistance; however, misconceptions regarding viral infections and the transmission of antibiotic-resistant bacteria were common. Gender, academic role, and medical family history were not statistically associated with knowledge level. Incorporating structured antibiotic stewardship education into dental curricula may help address the identified knowledge gaps.

## Introduction

Antibiotic resistance is a major global public health concern, driven in part by inappropriate antibiotic prescribing and use. Dentistry contributes to antimicrobial consumption, and inappropriate prescribing may expose patients to unnecessary adverse effects while contributing to the selection and dissemination of resistant bacteria [1]. Studies have reported substantial levels of non-guideline-concordant antibiotic prescribing in dental practice, highlighting the need for effective antimicrobial stewardship within dentistry [2]. Because dentists play an important role in antimicrobial prescribing, adequate knowledge of antibiotic indications, adverse effects, and resistance mechanisms is essential for safe and evidence-based clinical practice [3]. In such a dynamic environment, the dental profession may bear considerable responsibility; research has revealed troubling prescription rates that fall far from the standard accepted by the dental profession. Several studies have demonstrated that only 19% of antibiotics that were prescribed for serious dental problems were in accordance with professional guidelines [4,5]. The same applies to dental preventive antibiotic use, with US research showing that over 80% of antibiotics prescribed were clinically inappropriate [6,7]. The most commonly prescribed drugs for dental conditions are antibiotics and non-steroidal anti-inflammatory drugs (NSAIDs) [8,9].

Antibiotic resistance is a growing global public health concern, with inappropriate antimicrobial use contributing to the selection and dissemination of resistant organisms. Dentistry is an important component of antimicrobial stewardship because dentists frequently prescribe antibiotics for oral and dental conditions [10]. In India, inappropriate antibiotic use remains a concern, highlighting the importance of strengthening antimicrobial stewardship competencies during undergraduate dental education [11]. However, evidence regarding antibiotic resistance knowledge and misconceptions among dental trainees in regional Indian academic settings remains limited [12]. Therefore, the present study assessed knowledge and misconceptions regarding antibiotic use and antibiotic resistance among fourth-year dental students and clinical interns at Teerthanker Mahaveer Dental College and Research Centre, Teerthanker Mahaveer University, Moradabad, Uttar Pradesh, India.

## Materials and methods

Study design and setting

This cross-sectional study was conducted over four weeks, from January 15, 2026, to February 11, 2026, at Teerthanker Mahaveer Dental College and Research Centre, Teerthanker Mahaveer University, Moradabad, Uttar Pradesh, India. Fourth-year dental students and clinical interns were selected because they represent the senior stages of undergraduate dental education, when students have completed most of their foundational biomedical and pharmacological instruction and have substantial exposure to clinical dentistry. Including both groups allowed assessment of knowledge before and during clinical internship and provided an opportunity to examine whether progression from senior undergraduate education to clinical exposure was associated with differences in antibiotic-related knowledge. Third-year students were not included because they had comparatively less clinical exposure and were outside the predefined target population for this study.

Ethical considerations

Institutional ethical clearance was obtained from the Institutional Ethics Committee of Teerthanker Mahaveer Dental College and Research Centre before commencement of the study. All participants provided informed written consent electronically before participating in the survey. Participation was voluntary, and participants were informed that their responses would be collected and analysed confidentially. These samples were used for the study and for sample selection. The target groups were dental students at the upper level of their studies (fourth-year undergraduates and clinical interns). The population of all the individuals that could be available for the finite population was 130.

The target population consisted of 130 eligible fourth-year dental students and clinical interns. An initial minimum sample size of 98 participants was estimated for an infinite population. Because the available population was finite, the finite population correction (FPC) was applied using the formula,

$n_f = \frac{n}{1 + \left(\frac{n-1}{N}\right)}$

where n represents the initial sample size and N represents the total eligible population. With n=98 and N=130, the corrected minimum sample size was approximately 56 participants. A total of 130 eligible students were invited to participate, and 108 completed valid questionnaires and were included in the final analysis.

Inclusion criteria

Dental students at Teerthanker Mahaveer University in the fourth year or internship level of the course, during the data collection period, who were willing to provide informed consent were included in the study.

Exclusion criteria

Incomplete questionnaires, dental students who refused to participate, and non-dental students were excluded.

Data collection and questionnaire

The questionnaire (provided in the Appendices) was adapted from an instrument previously developed and validated among healthcare prescribers in Jordan. The items were reviewed for relevance and clarity in the context of undergraduate dental education and were administered in English, the language of instruction at the institution. However, the adapted questionnaire was not formally revalidated for the Indian dental student population in the present study. Therefore, the reliability, construct validity, and other psychometric properties of the adapted instrument in this population remain uncertain. This limitation should be considered when interpreting the findings and comparing them with studies using formally validated instruments.

Statistical analysis

The completed survey data were entered into a Microsoft Excel (Microsoft Inc., Redmond, WA) spreadsheet and then into the Statistical Package for the Social Sciences (SPSS) version 24.0 (IBM Corp, Armonk, NY) for statistical analysis. Descriptive statistics (frequencies and percentages) were used to gather response distributions and demographic characteristics. Inferential statistics, using Pearson's chi-square test, were employed to determine if there were relationships between basic knowledge parameters and the demographic characteristics (gender, academic year, family background). A p-value of less than 0.05 was chosen as the significance level.

## Results

Socio-demographic profile

Of the 130 eligible fourth-year dental students and clinical interns invited to participate, 108 completed the questionnaire and provided valid responses, yielding a response rate of 83.1%. All 108 completed questionnaires were included in the final analysis. Among participants, 72 (66.7%) were women and 36 (33.3%) were men. The cohort comprised 57 (52.8%) fourth-year undergraduate students and 51 (47.2%) clinical interns. A family member working in healthcare was reported by 52 (48.1%) participants, whereas 56 (51.9%) reported no such family background​ (Table 1).

**Table 1:** Socio-demographic profile of the study participants (N=108)

Characteristic	Category	n (%)
Gender	Female	72 (66.7)
Gender	Male	36 (33.3)
Academic role	Fourth-year undergraduate	57 (52.8)
Academic role	Clinical intern	51 (47.2)
Family medical background	Present	52 (48.1)
Family medical background	Absent	56 (51.9)
Total		108 (100.0)

Evaluation of knowledge and misconceptions

Conceptual gaps among the trainees were identified in the survey. The most notable was the large proportion of participants (n=71; 65.7%) who incorrectly reported that antibiotics could be used for viral infections, with only (n=33; 30.6%) correctly reporting this as false (Table 2).

**Table 2:** Participants' knowledge of antibiotic use and resistance (N=108)

Knowledge item	Correct response, n (%)
Antibiotics are not effective against viral infections	33 (30.6)
A full course of prescribed antibiotics must be completed	99 (91.7)
Unnecessary antibiotic use accelerates resistance	90 (83.3)
Antibiotics carry direct risks/side effects (e.g., colitis, allergies)	83 (76.9)
Resistant bacteria can spread from person to person	40 (37.0)
Each treated patient has an elevated localized risk of resistance	49 (45.4)

However, awareness of procedural compliance was very good. Among the respondents, 99 (91.7%) students considered it essential to take the full course of prescribed antibiotics, and 90 (83.3%) correctly noted that unnecessary use of antibiotics contributes to the rapid emergence of antibiotic resistance. Significant ambiguity occurred regarding epidemiological concepts, with only (40 (37.0%) students able to explain that resistant strains are horizontally transmitted from person to person, and fewer than half (n=49; 45.4%) able to say that each individual treated has an increased, localised risk of developing resistant microbes.

Bivariate association analyses

The response accuracy was cross-tabulated with demographic indicators, and chi-square tests were used to assess the differences among them with respect to knowledge variance. There was no association with any of the tested activities (Table 3).

**Table 3:** Pearson's chi-square test; statistical significance set at p<0.05.

Association tested	Groups compared	χ² statistic	p-value	Significance
Gender vs. viral indication misconception	Female vs. male	0.21	0.65	Not significant
Academic role vs. course completion awareness	Fourth-year undergraduate vs. clinical intern	0.1	0.75	Not significant
Family medical background vs. resistance drivers	Present vs. absent	0.91	0.34	Not significant
Academic role vs. transmission knowledge	Fourth-year undergraduate vs. clinical intern	1.32	0.25	Not significant

Gender vs. viral indication misconception

Knowledge proficiency did not seem to be determined by gender. No statistically significant differences were found for the point of greatest concern, namely, antibiotic activity against viral infections. The majority of participants were women (n=72; 66.7%), while male participants accounted for (n=36; 33.3%). There was no significant difference between senior undergraduates (fourth year) and clinical interns regarding their awareness of completing the prescribed antibiotic course, which was consistently good in both groups.

Family background vs. resistance drivers

No statistically significant benefit was seen from having a family member who was involved in a medical or its related fields.

Clinical exposure vs. epidemiological transmission knowledge

Worryingly, there was no improvement in basic public health concepts during the transition from didactic courses (fourth year) to clinical immersion (internship). There was no statistically significant association between the category of person (undergraduate vs intern) and correct responses concerning the horizontal transmission of resistant bacteria from person to person.

## Discussion

This cross-sectional study revealed a gap between the knowledge of the participants about the general principles of antibiotic use and their understanding regarding specific antimicrobial indications and the transmission of antimicrobial resistance (AMR). The vast majority of participants understood the need to adhere to the prescribed antibiotic regimen and that overuse of antibiotics is a driver of resistance. There were significant misconceptions about the effectiveness of antibiotics for treating viral infections and the spread of resistance bacteria. Antimicrobial resistance is considered a global public health threat by the World Health Organization (WHO) and rational antimicrobial use is an integral part of antimicrobial stewardship [12].

Antimicrobial use is a significant factor in primary care, both in terms of quantity and inappropriate prescription, and dentists make a significant contribution. The use of antibiotics has risen significantly from 2000 to 2015, and there was significant variation in the use of these drugs across different regions of the world [14]. There have also been studies which found inappropriate or non-guideline-concordant antibiotic prescribing in various settings [15,16]. Such results could be used to ground the competencies needed to enhance antimicrobial stewardship in undergraduate dental education.

One of the important results of the current study was that 71 (65.7%) participants believed that antibiotics are effective to treat viral infections. This finding indicates that there is a specific problem with indications for antibiotic use despite the high level of awareness around general antibiotic-use principles. Previous studies have shown that healthcare students and professionals are not always up to date on knowledge of antibiotics and that knowledge can differ significantly among the various aspects of antimicrobial stewardship [17]. This difference between bacterial and viral infections should be a large part of undergraduate antimicrobial stewardship education.

There is evidence of the impact of this misconception on clinical outcomes relating to the impact of unnecessary antibiotic exposure. Adverse reactions to antibiotic use, such as allergies and gastrointestinal effects, can occur and antibiotic exposure that is not needed can also lead to antimicrobial resistance [18,19]. Dental antibiotic prescriptions have also been linked to infection with *Clostridioides difficile*, suggesting that there might be a risk related to the inappropriate use of antibiotics in the dental practice setting [20]. According to evidence-based dental guidelines, definitive dental treatment is necessary for most pulpal and periapical conditions, whereas antibiotics should be used when systemic involvement or increased risk of systemic progression occurs [19]. Thus, knowledge of appropriate indications is an important component of safe and evidence-based dental prescribing.

The current study also found that the participants lacked knowledge about the transmission of AMR. As many as 40 participants (37.0%) correctly identified that resistant bacteria can be transmitted from person to person, and 49 (45.4%) correctly identified an elevated localised risk of antibiotic exposure. Based on these results, education should not only focus on the principles of antibiotic prescribing but also the epidemiology and spread of AR. Dental practice during COVID-19 has revealed shifts in the use of antibiotics, such as using more during times of limited definitive operative treatment [21]. This further highlights the need to adhere to evidence-based antimicrobial prescribing principles in different clinical situations.

Participants had generally high levels of awareness for the need to complete antibiotic courses (99, 91.7%) and that unnecessary antibiotic use leads to antibiotic resistance (90, 83.3%), but this was not seen for all indicators measured. These reactions contrasted with the significant misconception about viral infections indicated that overall awareness of antibiotic resistance may not be a marker for overall understanding of antimicrobial stewardship.

No statistically significant relations were detected between the following variables and the viral-infection misconception, family medical background and resistance-related knowledge, academic role and antibiotic-course awareness, and academic role and knowledge of transmission. These results indicate that there are no differences between the specific knowledge domains studied in this cohort for the demographic and academic-role variables selected. But, it is important to note that the lack of statistically significant relationships does not mean that clinical education has no effect on knowledge of antimicrobials. Other aspects of antimicrobial stewardship education such as previous exposure to antimicrobial stewardship education, access to antimicrobial stewardship prescribing guidelines, and content of the education were not evaluated.

Limitations

Several constraints limit the interpretation of this study's findings. First, the study was conducted using a cross-sectional design; thus, only a snapshot of participants' knowledge was obtained, and changes in knowledge or cause-and-effect relationships between demographics and antibiotic knowledge could not be assessed. Second, the study was conducted with a relatively small number of dental students (108) from a single academic setting, which may limit the generalizability of the results to dental students at other institutions or geographic areas. Third, knowledge was measured using self-reported responses to the questionnaire, which could have introduced recall, response, and social desirability biases. Finally, the analysis was restricted to selected knowledge items and demographic characteristics; therefore, other potentially relevant factors were not analysed (e.g., antimicrobial stewardship training previously received, experience with clinical antimicrobial prescribing, exposure to continuing education, and access to prescribing guidelines). Lastly, the study was based on knowledge and misconceptions and not on actual prescribing practices. This means that an incorrect answer cannot be taken as evidence of inappropriate antibiotic prescribing in the clinic. Studies conducted across multiple centres using widely used instruments and longitudinal designs, as well as examinations of real prescribing behaviour, would yield stronger evidence of dental students' antimicrobial stewardship competencies. Finally, the questionnaire was adapted from an instrument validated in a Jordanian healthcare population and was not subjected to formal psychometric revalidation in the present Indian dental cohort, which may affect the reliability and comparability of the measured knowledge outcomes.

## Conclusions

Despite high compliance with the basic principles of antibiotic dosing, this study suggests a significant lack of basic knowledge of antibiotics among senior dental students and interns. One of the notable gaps in the present cohort was the misunderstanding of viral infections and limited understanding of how resistant bacteria can propagate. These gaps were observed across students regardless of academic role and may warrant further strengthening of antimicrobial stewardship education.

## Appendices

Questionnaire

**Table 4:** Questionnaire on Knowledge and Misconceptions Regarding Antibiotic Use and Antibiotic Resistance

Question	Correct response
Antibiotics are not effective against viral infections.	FALSE
A full course of prescribed antibiotics must be completed.	TRUE
Unnecessary antibiotic use accelerates resistance.	TRUE
Antibiotics carry direct risks/side effects (e.g., colitis, allergies).	TRUE
Resistant bacteria can spread from person to person.	TRUE
Each treated patient has an elevated localized risk of resistance.	TRUE
